# The effect of extended continuous nursing strategy applied to patients with mild brain injury on their quality of life and self-efficacy

**DOI:** 10.3389/fsurg.2022.981191

**Published:** 2022-09-12

**Authors:** Li Zhang, Yanmei Ma, Jia Liu, Miao Cai, Wenqiu Zheng

**Affiliations:** ^1^ Department of Rehabilitation, The First Affiliated Hospital of Jinzhou Medical University, Jinzhou, China; ^2^ Department of Nursing, The First Affiliated Hospital of Jinzhou Medical University, Jinzhou, China; ^3^ General Gastroenterology II Ward, The First Affiliated Hospital of Jinzhou Medical University, Jinzhou, China

**Keywords:** continuous nursing, brain injury, mild, quality of life, self-efficacy

## Abstract

Postoperative rehabilitation of craniocerebral injury requires a long process and has many complications. In addition, patients with severe craniocerebral injury are usually accompanied by impaired nervous system function, which will affect the patients’ normal life and work in a period of time after surgery. Reasonable rehabilitation nursing plays an active role in restructuring central nervous system function and coordinating muscle and joint activities. Since the rehabilitation of cerebral trauma is a long process, how to ensure the patients to carry out limb and brain function as well as self-care ability and self-care skills according to the rehabilitation exercise plan and intervention measures formulated before discharge has aroused hot debate. This study analyzed the impact of out-of-hospital continuous nursing strategy applied to patients with mild cerebral trauma on their quality of life and self-efficacy level.

## Introduction

Traumatic brain injury is a common type of surgical system disease in clinical practice. As the brain is a very important organ in the human body, it can dominate the various acts of the body and guide the body to make the corresponding response under the instruction. Once damaged, it will greatly affect the quality of daily life of individuals (1). Patients with mild traumatic brain injury are a relatively special group. On the one hand, there is an individual's self-consciousness, they can feel physical pain and have a certain degree of awareness of their own illness. On the other hand, the lack of self-confidence in the ability to care will further induce individuals to experience negative emotional perceptions such as anxiety and depression (2). So how to effectively connect the nursing during hospitalization and self-care outside the hospital is a major research topic in the field of nursing.

In order to meet the rehabilitation needs of patients after discharge, this study aims to provide a correct self-care channel for the patient's condition management outside the hospital from the perspective of the out-of-hospital continuous nursing strategy, effectively mobilize the individual's internal subjective initiative, enable the patient to gradually internalize the specific nursing knowledge score, and guide the individual to actively respond to the out-of-hospital condition. All kinds of sudden life events can help individuals master a healthy life philosophy and make the disease develop in a benign direction (3, 4). This intervention program pays more attention to the independent behavior of individuals, and treats the hospitalization and discharge phases of patients as a complete process for management, providing patients with good out-of-hospital care, thereby promoting all-round outcomes of the disease and reducing the long-term sequelae of traumatic brain injury (5, 6). The out-of-hospital continuous nursing strategy puts forward higher requirements for both nurses and patients. The author intends to design a randomized and controlled scientific research idea to further analyze the clinical effect of out-of-hospital continuous nursing strategy applied to patients with mild traumatic brain injury. The research results are now reported as follows:.

## Research objects and methods

### Research objects

Our hospital admitted and treated 120 patients with mild traumatic brain injury from September 2018 to November 2019. Using the digital table as the basis for grouping, the research subjects of 60 cases who met the inclusion conditions were named as the research group and the control group. Using the digital table as a grouping tool, the subjects who met the inclusion requirements were named as the research group and the control group, with 42 cases in each group.

### Inclusion requirements

The patient underwent cranial magnetic resonance and CT examination, combined with his physical symptoms and signs, and was diagnosed with mild traumatic brain injury. diagnostic criteria for the disease; The patient did not have cognitive abnormalities; There are no abnormalities in hearing and vision; have basic verbal communication skills; know the content of this research and sign the consent form; There is no hemolysis and coagulation abnormality; there is no autoimmune system disorder.

### Methods

#### Control group

Routine nursing measures were carried out in this group of patients. Based on the physiological comfort that the patient can perceive on the current body surface, the nurse effectively adjusts the temperature and humidity in the department to ensure that the patient can feel comfortable physically and mentally. Instruct the family to provide the patient with as much food as possible in the meal, high-quality protein, vitamins and low-fat foods. Make the hospital bed clean and fresh; dynamically monitor the patient's current physical symptoms, and notify the doctor in time and take symptomatic treatment once any abnormality is found. And guide individuals to vent their inner fears, anxiety and panic and other negative emotions.

#### Research group

The patients in this group carried out the out-of-hospital continuous nursing strategy on the basis of the routine nursing measures of the control group. ① Draw up continuous nursing strategies: The responsible nurse as the initiator, integrates the rest of the nurses in the undergraduate room to form a continuous nursing team with a total of 3 members, of which 1 has an intermediate title and the other 3 have a primary title. The responsible nurse as the leader, organizes a joint discussion among all members, using “mild traumatic brain injury” and “continued nursing” as the key words to search and select domestic well-known databases such as “China Knowledge Network”, “VIP” and “Wanfang”, from which references with high evidence-based levels were selected. All the team members discussed the selected references again, and finally clarified the nursing intervention measures needed for this study. After that, practical skills training was organized for the clear nursing intervention strategy process, and the training time was limited to 8 h. Furthermore, the theoretical cognition and practical skills assessment will be implemented for all team members, and only those who pass both of the assessment objects can enter the next stage of nursing intervention; If one of the assessment results fails, they will need to undergo training again until the assessment results are qualified. ② Pairing patients and their families: Distribute a piece of A4 white paper to the patient, and ask them to list their immediate family members on the A4 white paper, and require that the listed immediate family members live for at least 8 weeks or more. Nurses pair patients with their families and provide them with a relatively private and undisturbed environment for subsequent cognitive interventions. First, the nurse distributed white paper and black pens to both parties, informing both parties in advance that they need to internalize the key points that appeared in this health education. The time for nurses' health education is limited to 20 min, and the content involves the main points of out-of-hospital self-care for mild traumatic brain injury. After finishing the mission, the nurse asked both parties to exchange A4 sheets of recorded points, and asked both parties to retell the important knowledge points understood and internalized this time. Nurses made multi-dimensional corrections for the missing knowledge points and cognitive biases existing on both sides, thereby strengthening both sides' further cognition of self-care behavior outside the hospital. The whole process of the paired cognitive intervention was recorded by a special person with a mobile phone, and the electronic audio was copied to both parties, and both parties were required to study and strengthen them repeatedly 30 min before going to bed that night. ③ Out-of-hospital real-scenario simulation exercise: Nurses propose specific scripted scenarios based on the situation of cognitive education and common self-care situations that may occur outside the hospital. The scenario requires that the patient's out-of-hospital self-care content be included, such as: “Now, you suddenly feel pain in your brain, what is the first thing you think of?”, “What is your current emotional state?”, “What measures will you take to deal with it?” etc. Give patients and their families 3 min to immerse themselves in situational awareness and think in combination with the knowledge system they have mastered. In the follow-up role-playing process, the nurses observe the patient's current behavioral performance and mental and emotional state in an all-round way, and then a dedicated person uses a mobile phone to record the whole process. The nurse then decomposes the content of each role-playing situation one by one in the form of video playback, and corrects the cognitive deficits reflected by the patient's behavior, thereby strengthening the individual's internalization and understanding of the relevant knowledge system, so that it can be transformed into own current care behavior. The role-playing time is limited to 10 min. Patients were required to review the recorded specific video 20 min before going to bed that night, and recall according to the nurse's comments, correct their own misunderstandings, so as to continuously consolidate and strengthen the corresponding role-playing situations. ④ Sharing of experience in the symposium: The nurses followed up the patients after discharge, and asked to come to the hospital for a symposium every 2 weeks. During the symposium, each patient was given 2 min to come to the stage to share their experiences and experiences in the past 2 weeks. At the same time, each patient was asked to share their own experiences and insights in text or voice after the symposium way to record, so as to ensure that you can record your own status in real time. The format of each symposium is controlled within 1 h. Afterwards, a WeChat group was established for all patients, and all patients were required to join the group, and everyone was required to share their experiences related to this symposium in text or voice, so as to improve the initiative and enthusiasm of patients.

### Observation items

① The patients in the two groups were evaluated by the SF-36 quality of life scale before the intervention and at the 4th weekend after the intervention. The scale contains a total of 7 dimensions, and the score of each dimension is 0–100 score. It indicates that the quality of life level of the corresponding dimension is better (7).

② Both groups of patients received self-efficacy questionnaires before the intervention and at the 4th weekend after the intervention. The questionnaire included 5 dimensions (basic knowledge of the disease, cognition of the hazards of adverse factors, treatment knowledge, self-management knowledge, and complication prevention knowledge). Each dimension is divided into 3 levels, namely poor, good and excellent, and a 3-level scoring method (1 ∼ 3 score) is adopted. The higher patient's score means the better the level of self-care (8).

### Statistical methods

All the data that met the inclusion criteria were entered into SPSS19.0 software for processing, and the quantitative data were statistically described by the mean and standard deviation, and the t-test was used for comparison between groups; Qualitative data were described by rate, and chi-square test was used for comparison between groups; The rank data were tested by rank sum test. When *P *< 0.05, the difference was statistically significant.

## Results

### General information

There was no significant difference in the baseline data of the two groups of patients after statistical comparison (*P *> 0.05). As shown in Table 1.

**Table 1:** Comparison of baseline data of two groups of patients.

Group	*N*	Gender (male/female, *n*)	Age (year)	Cultural level		
				Primary school	Middle school	College
Research group	60	42/18	41.9 ± 2.7	16	32	12
Control group	60	44/16	42.1 ± 2.8	17	34	9
*χ^2^*/*t*	—	0.164	0.153		0.519	
*P*	—	0.685	0.878		0.771	

### Comparison of the quality of life scores of the two groups before and after the intervention

After the intervention, the scores of the SF-36 quality of life scale in the research group were higher than those in the control group, and the difference was statistically significant (*P *< 0.05). As shown in Table 2.

**Table 2:** Comparison of quality of life scores between the two groups before and after intervention (score).

Indexes	Before intervention				After intervention			
	Research group (*n* = 60)	Control group (*n* = 60)	*t*	*P*	Research group (*n* = 60)	Control group (*n* = 60)	*t*	*P*
Physical function	62.4 ± 6.9	62.6 ± 7.0	0.673	>0.05	82.1 ± 10.3	70.4 ± 7.4	13.271	<0.05
Body role	63.6 ± 6.9	64.0 ± 7.0	0.901	>0.05	81.4 ± 10.6	71.5 ± 8.4	9.367	<0.05
Muscle pain	65.2 ± 4.6	65.7 ± 5.0	0.457	>0.05	83.2 ± 8.9	72.7 ± 8.3	7.267	<0.05
General health status	68.7 ± 6.2	69.0 ± 6.3	0.678	>0.05	79.4 ± 8.7	73.1 ± 7.1	9.031	<0.05
Social function	66.4 ± 4.6	66.6 ± 4.8	0.901	>0.05	83.9 ± 9.4	72.6 ± 5.2	15.378	<0.05
Vitality	68.1 ± 5.2	68.3 ± 5.4	1.177	>0.05	84.3 ± 10.6	73.5 ± 5.8	13.278	<0.05
Mental health	62.1 ± 6.0	61.9 ± 5.8	0.453	>0.05	81.1 ± 8.8	71.8 ± 6.9	16.268	<0.05
Emotional role	65.5 ± 5.7	65.7 ± 5.8	0.789	>0.05	82.7 ± 9.6	72.6 ± 7.1	9.013	<0.05

### Comparison of self-efficacy levels before and after intervention in the two groups

After intervention, the scores of basic disease knowledge, adverse factor perception, treatment knowledge, self-management knowledge, and complication prevention knowledge in the research group were higher than those in the control group, and the difference was statistically significant (*P *< 0.05). As shown in Table 3.

**Table 3:** Comparison of self-efficacy levels before and after intervention in the two groups (score).

Indexes	Before intervention				After intervention			
	Research group (*n* = 60)	Control group (*n* = 60)	*t*	*P*	Research group (*n* = 60)	Control group (*n* = 60)	*t*	*P*
Basic knowledge of disease	0.8 ± 0.1	0.8 ± 0.1	0.890	>0.05	2.2 ± 0.5	1.3 ± 0.3	13.214	<0.05
Adverse factor risk perception	1.1 ± 0.3	1.0 ± 0.2	1.027	>0.05	2.3 ± 0.6	1.4 ± 0.3	9.367	<0.05
Treatment knowledge	1.2 ± 0.2	1.3 ± 0.3	0.916	>0.05	2.2 ± 0.6	1.3 ± 0.3	10.673	<0.05
self-management knowledge	1.0 ± 0.1	1.1 ± 0.2	0.675	>0.05	2.4 ± 0.4	1.5 ± 0.3	7.367	<0.05
Complication prevention knowledge	1.2 ± 0.2	1.1 ± 0.1	0.589	>0.05	2.4 ± 0.7	1.6 ± 0.4	15.372	<0.05

## Discussions

Patients with mild traumatic brain injury are prone to symptoms such as headache, dizziness, and irritability, which significantly reduce the quality of life of patients (8). Due to the long course of the disease, if the patient's personal self-care behavior is not properly managed, under the stimulation of external adverse factors, the patient will be induced to relapse (9). This will not only increase the medical costs, but also increase their physical pain. Since patients usually come to the hospital for treatment in the acute stage, they need to face the distress of discharge after the condition is controlled (10). And after discharge, it also involves the level of personal self-care behavior. If the patient's self-care ability is unreasonable, it will indirectly affect the patient's health status (11). So, improving the self-management ability of patients after discharge is conducive to the overall prognosis and prognosis of their diseases.

In terms of improving the self-management ability of individuals, traditional nursing intervention methods usually adopt the method of health education to let patients understand the theoretical knowledge system of disease occurrence, development, prognosis and outcome (12). However, because the nursing intervention strategy focuses more on the “cramming” instillation of medical knowledge, while ignoring the internal demands of patients at the mental and psychological level, the intervention effect often fails to meet expectations, and it also causes patients to appear after discharge due to past bad behaviors (13). Therefore, under this background, some scholars have proposed a care model based on the concept of continuity (14). This model effectively connects the life and daily care in the hospital and the hospital, starting from the theoretical cognitive level and situational role-playing measures. Strive to let patients have an independent cognition of the disease, and consciously practice the corresponding self-protection behaviors, and achieved considerable clinical results.

The results of this survey showed that the quality of life scores of the patients in the research group after intervention were higher than those in the control group, and the difference was statistically significant (*P *< 0.05). This indicates that the out-of-hospital continuous nursing strategy applied to patients with mild traumatic brain injury can help improve the quality of life of patients in all dimensions. The reasons for this result are closely related to the following factors. ① The determination of intervention plan is the specific presentation of evidence-based medicine model (15). At present, medicine has changed from the traditional empirical medicine to the evidence-based medicine. Therefore, in this study, based on the current internal demands of patients and combined with their specific clinical actual situation, the most cutting-edge theoretical and practical skills were obtained through literature retrieval on databases such as Wanfang, HowNet and VIP, thus guiding clinical practice and enabling patients to obtain the optimal diagnosis and care. On this basis, standardized and standardized training is also carried out for the team members, so that they can master and understand the standardized care mode for patients with traumatic brain injury, and finally improve the management mode of the disease by nurses. ② Patient-family paired cognitive intervention is the premise and guarantee to ensure continuous post-hospital care for patients (16). Only a correct cognitive system can ensure that patients consciously practice correct and scientific behaviors, thereby reducing the incidence of adverse events, avoiding risk factors, and making them face the disease with a more peaceful mind. The traditional cognitive intervention model has not yet started from the patient's subjective level, and more is to provide patients with passive theoretical knowledge points, which cannot ensure that patients can better internalize and absorb relevant knowledge content (17). Under this opportunity, we started from the patients’ closest relatives to provide them with appropriate clinical diagnosis and care, so as to ensure the holistic prognosis and outcome of the patient's condition. The patients and their families are organized into teams, so that the patients and their families can learn from each other and make up for the cognitive deficiencies and deficiencies of each other, thereby improving the cognitive level of both sides to the greatest extent in a short time (18). In addition, when the family members also have some understanding of the relevant knowledge content, it provides a guarantee for the construction of an integrated and harmonious atmosphere of the family, and avoids the occurrence of family disputes. In addition, in the process of cognitive intervention, the characteristics of the Ebbinghaus forgetting curve are also introduced. The patient not only has a preliminary understanding of the relevant knowledge score during the study, but also accepts the intervention explained by the two parties after the end. Before going to sleep, recall the content of relevant theoretical knowledge. With the help of the construction of the “recall-memorizing-contemplation” model, it can ensure that they understand the relevant knowledge to the greatest extent. ③ The role-based situational play is based on the patient's mastery of relevant theoretical knowledge (19). After the patient has a comprehensive understanding of the relevant knowledge, behavior management training is required. Effectively avoid and reduce the incidence of adverse events. When the patient thinks about a specific situation, it can make the patient enter into the relevant situation that he may encounter in the future in advance face (20). In addition, when the patient performs specific role-playing, in fact, the relevant theoretical knowledge points that have been mastered before are fully retrieved and put into practice, and finally the internalization and absorption of relevant theoretical knowledge can be achieved. At the same time, nurses use mobile phones to record patients' role-playing situations, guide them, and ask them to observe and learn repeatedly, which can ultimately achieve good behavior management. ④ The sharing of experience in the symposium is a platform that brings patients together, builds a platform between the hospital and patients, and enables patients to communicate with each other. During the communication process, they can re-examine their self-care performance during this period of time, so as to help its architecture system and comprehensive theoretical knowledge system (21). In addition, after the communication is over, the patient is allowed to record the current experience in text and audio, so as to ensure that the patient can further memorize the relevant content. Furthermore, with the help of WeChat Moments, it can provide a 24-hour platform for communication between patients, which is beneficial for both parties to share and exchange relevant theoretical knowledge in real time. Therefore, with the smooth implementation of the above intervention measures, the self-efficacy level of patients can be effectively improved, which is beneficial to the improvement of personal quality of life. Therefore, the results of this survey show that the quality of life of patients in the research group after intervention is higher than that in the control group., the difference was statistically significant (*P *< 0.05).

In conclusion, the out-of-hospital continuous nursing strategy applied to patients with mild traumatic brain injury can improve the self-efficacy level of patients, and can improve their quality of life and nursing satisfaction, which is worthy of further promotion in clinical practice. However, the sample sources of this study are concentrated, the number of samples is small, and it is a single-center study. The follow-up needs large-scale and multi-center research to further demonstrate the research results.

## Data Availability

The original contributions presented in the study are included in the article/Supplementary Material, further inquiries can be directed to the corresponding author/s.
